# The Exocrine Differentiation and Proliferation Factor (EXDPF) Gene Promotes Ovarian Cancer Tumorigenesis by Up-Regulating DNA Replication Pathway

**DOI:** 10.3389/fonc.2021.669603

**Published:** 2021-05-10

**Authors:** Yangjiong Xiao, Yunxin Lai, Yang Yu, Pengcheng Jiang, Yuhong Li, Chao Wang, Rong Zhang

**Affiliations:** ^1^ State Key Laboratory of Respiratory Disease, National Clinical Research Center for Respiratory Disease, Guangzhou Institute of Respiratory Health, the First Affiliated Hospital of Guangzhou Medical University, Guangzhou, Guangdong, China; ^2^ Department of Obstetrics and Gynecology, Shanghai Fengxian District Central Hospital, Southern Medical University, Shanghai, China; ^3^ Department of Microbiology, Institute of immunology, Perelman School of Medicine, University of Pennsylvania, Philadelphia, PA, United States; ^4^ Shanghai Key Laboratory of Regulatory Biology, Institute of Biomedical Sciences, School of Life Sciences, East China Normal University, Shanghai, China; ^5^ Department of Gynecology, Changzhou Second People’s Hospital Affiliated to Nanjing Medical University, Changzhou, China; ^6^ Department of Gynecology, The International Peace Maternity & Child Health Hospital, The China Welfare Institute, Shanghai Jiaotong University, Shanghai, China

**Keywords:** EXDPF, PPDPF, ovarian cancer, DNA replication, target therapy

## Abstract

The Exocrine Differentiation and Proliferation Factor (EXDPF) gene could promote exocrine while inhibit endocrine functions. Although it is well known that ovary is an endocrine organ, the functions of EXDPF in ovarian cancer development is still unknown. This study demonstrated that EXDPF gene is significantly higher expressed in ovarian tumors compared to normal ovarian tissue controls. EXDPF DNA amplification was exhibited in lots of human tumors including 7.19% of ovarian tumors. Also, high expression of EXDPF positively correlated with poor overall survival (OS) of ovarian cancer patients. EXDPF expression could be universally detected in most epithelial ovarian cancer cells (SKOV3, IGROV1, MACS, HO8910PM, ES2, COV362 and A2780) tested in this study. Knock-down of EXDPF by siRNA delivered by plasmid or lentivirus largely inhibited ovarian cancer cells, IGROV1 and SKOV3 proliferation, migration and tumorigenesis *in vitro* and/or *in vivo*. Knock-down of EXDPF sensitized SKOV3 cells to the treatment of the front-line drug, paclitaxel. Mechanism study showed that EXDPF enhanced DNA replication pathway to promote ovarian cancer tumorigenesis. In conclusion, this study demonstrated that EXDPF could be a potential therapeutic target as a pro-oncogene of ovarian cancer.

## Introduction

Ovarian cancer has the highest mortality rate among gynecological cancers with a five-year overall survival (OS) rate remains as low as 30% - 40% in these two decades (1–3). The estimated new cases and death of ovarian cancer worldwide in 2020 was 313,959 and 207,252, respectively (4). This means the ratio of mortality to incidence of ovarian cancer as high as 0.660. There are several factors that responsible for the high mortality of ovarian cancer. Firstly, the symptoms of ovarian cancer at early stages are inapparent, which causes about 2/3 of patients at a late or advanced disease stage at diagnosis (5). At advanced disease stages, tumors are generally disseminated or metastasized to multiple organs especially to those in the abdominal cavity (6). Tumor metastasis makes it very hard to remove all tumor nodes by surgery, which often causes recurrence of cancer (7). Secondly, about 2/3 of ovarian cancers will eventually develop resistance to platinum, a kind of front-line chemotherapeutic drug against ovarian cancer (8). Thirdly, there are lack of novel therapies that could essentially improve OS of ovarian cancer patients. Take the inhibitors targeting poly ADP ribose polymerase (PARP) and immuno-therapy strategies targeting PD-1 and/or PD-L1 for examples. Although, PARP inhibitors, both FDA approved and under clinical trials, could increase 3 to 25 months of median progression-free survival (PFS) of ovarian cancer patients with a better response in BRCA mutated patients in clinical trials (9–11), less efficacy of PARP inhibitors on OS of ovarian cancer patients could be observed (12). Currently, three PARP inhibitors, Olaparib, Rucaparib and Niraparib, are approved by U.S. FDA to treat ovarian cancer patients. Generally, these PARP inhibitors could only increase 2% - 4% of OS, which is just about 2-5 months (13–15). Fortunately, recent report showed that Olaparib, as the maintenance treatment could improve 12.9 months of OS in BRCA mutated patients with platinum-sensitive relapsed ovarian cancer compared to placebo control (16). It is highly valuable to detect the effect of Olaparib treatment on platinum-resistant patients as most of ovarian cancer patients could derive platinum resistance. However, the OS improvement by Olaparib treatment in relapsed platinum-resistant ovarian cancer patients is still unclear. The efficacy of immunotherapies in ovarian cancer was very week, which may be associated with high immunosuppressive tumor microenvironment (17). Although anti-PD-1 and/or anti-PD-L1 antibody treatments rise in these few years, there is still no solid conclusion of the effect of these immunotherapies on ovarian cancer patients. However, the ~ 6 month improvement on the progression free survival (PFS) duration of these immunotherapies is still very weak (18, 19). The worse situation is that only 6% to 15% of ovarian cancer patients respond to PD-1 and/or PDL-1 blockade therapies (18, 19).

Better understanding of the tumorigenesis mechanisms is the critical factor to develop novel therapeutic strategies for high improvement of OS of ovarian cancer patients. The few understanding of ovarian cancer tumorigenesis is largely due to very high heterogenicity of ovarian cancer. Based on histopathology, ovarian cancer could be divided into epithelial ovarian cancer, germ cell cancer, sex cord-stromal tumor and metastatic tumors, usually arise from endometrium, breast, colon, gastric and cervical cancers. Epithelial ovarian cancer consists of 85% of ovarian cancers (20). As to epithelial ovarian cancer, it also could be divided into 5 subtypes of cancers that are high grade serous carcinoma (HGSC), low grade serous carcinoma, mucinous carcinoma, endometrioid carcinoma and clear cell carcinoma. HGSC consists of 75% of epithelial ovarian cancer and has the highest mortality rate among epithelial ovarian cancers. The five-year OS of HGSC is only 25% (21). The high genetic complexity and heterogenicity of epithelial ovarian cancer are mainly exhibited by different subtypes of epithelial ovarian cancer with different genomics, epidemiology and histopathological alterations (22). Taking gene mutation as an example, TP53, BRCA1/2, ATM, CSMD3, NF1, CDK13 and RB1 gene mutations usually occur in HGSC (23, 24). And the mutation rate of TP53 in HGSC reaches as high as 96% (25). While KRAS, BRAF, ARIDIA, PIK3CA, PTEN, and CTNNB1 gene mutations usually occur in other subtypes of epithelial ovarian cancers. For example, usually, low-grade serous carcinoma has ERBB2, BRAF and KRAS mutations (26), mucinous carcinoma has KRAS mutation (27), clear cell carcinoma has ARIDIA and PIK3CA mutations, and endometrioid cell carcinoma has CTNNB1, PTEN and PIK3CA mutations (28). Taking protein expression levels as an example, Martin et al. simultaneously detected the expression levels of 21 tissue-specific associated proteins in HGSC, mucinous carcinoma, clear cell carcinoma, and endometrioid carcinoma subtypes. They showed that the expression levels of 20 out of 21 of these proteins were significantly different among the subtypes (29). These previous studies strongly show that the genetic complexity and heterogenicity of epithelial ovarian cancer is very high.

The ovary is an endocrine and a terminally differentiated organ. The occurrence and development of epithelial ovarian cancer are closely related to the ovarian endocrine system. Studies have shown that the expression level of estrogen receptor (ER) and progesterone receptor (PR) in epithelial ovarian cancer are usually variable. And high expression of ER or PR is favorable to the prognosis of several subtypes of ovarian cancer (30). Except of ER and PR, endocrine associated genes are less studied in ovarian cancer. To elucidate novel characteristics associated with endocrine system in ovarian cancer, this study used high throughput genome mRNA sequencing to screen the mRNA expression profile of epithelial ovarian cancer. Our data showed that EXDPF, also named Pancreatic Progenitor Cell Differentiation and Proliferation Factor (PPDPF), was significantly higher expressed in ovarian tumors compared to the ovarian normal tissues from the same patients. The amino acid length of EXDPF is 114, and the protein molecular weight is 11.78 kDa. Gene Ontology (GO) annotation shows that the functions of EXDPF are associated with cell differentiation and exocrine pancreas development, which is mainly based on a previous study conducted in zebrafish (31). In zebrafish, EXDPF has been shown to promote the growth and differentiation of pancreatic exocrine glands while inhibit the growth and secretion function of pancreatic endocrine glands (31).

The role of EXDPF in cancers, especially in ovarian cancer, is still unclear. By knock-down of EXDPF expression, this study showed that EXDPF promoted ovarian cancer cell proliferation and migration in cell cultures, and ovarian cancer tumorigenesis and metastasis in mouse models. The underlying mechanisms of promoting tumorigenesis by EXDPF are associated with enhancing DNA replication signaling pathway.

## Materials and Methods

### EXDPF Knock-Down

shRNA plasmid for EXDPF knock-down (shEXDPF, cat# sc-105160-SH) and the control plasmid (shCON, cat# sc-108060) were bought from Santa Cruz (Dallas, TX, USA). For knock-down of EXDPF in IGROV1 cells, shEXDPF were delivered into IGROV1 cells at the presence of Polyethyleneimine (PEI, cat#23966-1, Polysciences, Warrington, PA, USA) as a transfection adjuvant. Twenty-four hours after transfection, cells were treated with 1.0 μM puromycin for 5 days. IGROV1 cells transferred with control plasmid were used as the vehicle negative control. Before experiments, EXDPF knock-down or control IGROV1 cells were cultured for 3 to 5 days without puromycin.

Lentivirus expressing shRNA targeting EXDPF were used for knock-down of EXDPF in SKOV3 cells. For lentivirus packaging, 293T cells were co-transfected with shCON or shEXDPF plasmid combining with the two packaging plasmids psPAX2 and pMD2.G at the present of PEI. Cell culture supernatants containing target virus were collected at 48 h and 72 h after transfection for virus collection. Lentivirus expressing shCON (LT-CON) or shEXDPF (LT-EXDPF) were used for infection of SKOV3 cells. Forty-eight hours after infection, SKOV3 cells were treated with 1.0 μM puromycin for a week. LT-CON or LT-EXDPF SKOV3 cells were cultured for 3 to 5 days without puromycin before experiments.

### RNA Sequencing

Three pairs of ovarian tumor tissues and the normal ovarian tissues from the same patients of epithelial ovarian cancer (numbered CZ001, CZ003 and CZ004) were derived from primary debulking surgery (PDS). Specifically, normal ovarian tissues were derived from tumor-adjacent tissues or the contralateral normal ovary. Tissues were kept in Nalgene tubes (ThermoFisher Scientific, #5012-0012) containing MACS tissue storage solution (Miltenyi Biotec, #130-100-008) and frozen in liquid nitrogen for storage. All patients have epithelial ovarian cancers and the characteristics including tumor stages and ages of patients are shown in **Supplemental Table S1**. Total RNA was extracted using TRIzol reagent (Thermo Fisher SCIENTIFIC, MA, USA) and RNA sequencing libraries were prepared using an Illumina Standard library preparation kit as our previous study (32). RNA sequencing was conducted by Shanghai OE Biotech Co., Ltd (Shanghai, China) using the Illumina HiSeqTM 2500 platform. mRNA expression values were normalized as fragments per kilobase of transcript per million mapped reads (FPKM). Statistical analysis was calculated using the DESeq Software Package (bioconductor.org), and *P* < 0.05 was considered statistically significant. KEGG pathway enrichment analysis was performed to detect the most affected pathways by knock-down of EXDPF. This RNA sequencing raw data could be accessed through National Center for Biotechnology Information (NCBI) at Sequence Read Archive (SRA) suing submission number SUB4998463.

### qRT-PCR

Both tumor tissues and normal ovarian tissues from 8 epithelial ovarian cancer patients (numbered CZ001, CZ003, CZ004, CZ008, CZ009, FX002, HFZ002 and HFZ003) as mentioned above and several epithelial ovarian cancer cells (A2780, COV362, ES2, HO8910PM, MACS, IGROV1, SKOV3, SKOV3-LT-CON and SKOV3-LT-EXDPF) were used for total RNA extraction using TRIzol reagent (Thermo Fisher). Specific clinical characteristics of patients are shown in **Supplemental Table S1**. cDNA was transcribed using a Superscript III Reverse transcriptase kit (Life Technologies) and used to measure the expression levels of target genes by SYBR Green Real-time PCR master mix kit (Takara, Shiga, Japan) on a 7900HT machine (Applied Biosystems, Foster City, CA, USA). The primer pairs used for amplifying corresponding genes were listed in **Supplemental Table S2**. GAPDH served as a housekeeping gene control. Relative mRNA expression levels of target genes were calculated by dividing Ct value of target genes by Ct value of GAPDH. mRNA expression levels in normal ovarian tissues or SKOV3-LT-CON control cells were normalized to 1.0.

### Detection of EXDPF mRNA Expression in Tumors and Normal Tissues in Database

The Human Protein Atlas online database (https://www.proteinatlas.org/) was used to compare EXDPF mRNA expression in 17 kind of human tumors and 36 kind of human normal tissues. The mRNA expression data in tumors was indexed from TCGA database, while in normal tissues was indexed from GTEx database. The gene name PPDPF or EXDPF could be used as search parameter in the searching toolbar and all other parameters were set with default.

### EXDPF DNA Alteration Analysis

EXDPF DNA alterations such as amplification, mutation and deletion in different kind of tumors were analyzed in cBioPortal online database (https://www.cbioportal.org/) by searching its gene name PPDPF in Quick Search toolbar and other parameters were set with default.

### Relationship Between EXDPF Amplification and Ovarian Cancer Patient Prognosis

Kaplan Meier-plotter online dataset of ovarian cancer database (http://www.kmplot.com/analysis/index.php?p=service&cancer=ovar) was used to analyze the relationship of EXDPF expression and overall survival (OS) of ovarian cancer patients. This online database was generated using gene expression data and survival information of 1287 ovarian cancer patients downloaded from Gene Expression Omnibus and The Cancer Genome Atlas (Affymetrix HG-U133A, HG-U133A 2.0, and HG-U133 Plus 2.0 microarrays) by Balázs Gyorffy and colleagues in 2012 (33). The probe 227994_x_at of PPDPF gene was used for analysis. Auto select best cutoff was chosen as the cut-off value parameter and data of patients at Stage 2, 3 and 4 that represented the late stage patients were selected for analysis.

### Western Blot Analysis

To detect the protein levels of EXDPF in control and EXDPF knock-down cells, total proteins were derived by lysing cells with RIPA buffer containing inhibitors of protease and phosphatase (Beyotime, Jiangsu, China). BCA protein assay kit (ThermoFisher Scientific) was used for detection of protein concentrations. Sixty μg total proteins were analyzed using electrophoresis on 10% SDS-PAGE gels followed by blotting onto polyvinylidene fluoride (PVDF) membranes. Anti-EXDPF antibody (#19912-1-AP, Proteintech, Wuhan, China) and anti-GAPDH (#AP0063) (Bioworld Technology, MN, USA) antibody were used for detecting protein expression of EXDPF and GAPDH, respectively. A secondary antibody, goat anti-rabbit IgG (#926-32211, LI-COR) labeled by IRDye 800CW, was used for luminescence by Image Studio Version 5.2 on an Odyssey CLx infrared imaging system (LI-COR).

### Cell Proliferation Assay

A total of 1 × 10^3^ control or EXDPF knock-down SKOV3 cells in a volume of 200 μl culture medium (RPMI-1640 containing 10% FBS, 100 U/ml penicillin and 100 µg/ml streptomycin.) were seeded into 96 well plates. And 1 × 10^5^ control or EXDPF knock-down IGROV1 cells in 2 ml culture medium were seeded into 6 well plates. Cells were detached by trypsin and counted using a hemocytometer every day for up to 5 to 7 days.

### IC_50_ Detection

A total of 1 × 10^3^ control or EXDPF knock-down SKOV3 cells in a volume of 100 μl culture medium were seeded into 96 well plates and cultured overnight. Cells were treated for another 72 h with paclitaxel at serial diluted concentrations from 10,000 nM to 4.57 nM. Cell viability was measured by 3- (4,5-dimethylthiazol-2-yl)- 5- (3-carboxymethoxyphenyl)- 2- (4- sulfophenyl)- 2H-tetrazolium (MTS) using a Cell Proliferation Assay kit (#G5430, Promega, Wisconsin, USA). Untreated cells were used as 100% viability controls and 50% inhibition concentration (IC_50_) values were calculated.

### Colony Growth Assay

A total of 1 × 10^3^ control or EXDPF knock-down cells (IGROV1 or SKOV3) in a volume of 2 ml culture medium were seeded into 6 well plates. After culturing for 10 days in a 5% CO_2_ incubator at 37°C, cells were fixed with 4% paraformaldehyde and stained with 2% crystal violet. Cell colonies were photographed and counted as a clone based on containing more than 50 cells.

### Cell Migration Assay

Cell migration assay was performed as described in our previous publications (32, 34). Briefly, polycarbonate membrane filter with 8 μm pore-transwells (Costar Group, DC, USA) were inserted into 24 well culture plates. A total of 1 × 10^5^ cells were resuspended in 200 μl RPMI-1640 medium without FBS, and seeded into a transwell. Each well of the 24 well plates was filled with 700 μl culture medium. Cells were cultured for another 18 h and fixed with 4% paraformaldehyde follow by staining with 2% crystal violet. Non-migrating cells that remained in the chamber were scraped off with cotton swabs. Migrated cells stuck to the bottom of the transwell membrane were photographed and counted at five random fields under a microscope with a magnification of 200-fold.

### Cell Cycle Assay

Control or EXDFP knock-down SKOV3 cells (1 × 10^5^/ml) treated with or without various concentrations of paclitaxel were seeded into 6 well plates in a volume of 2 ml culture medium and cultured for 24 h. Cells were harvested, washed and fixed with 70% ethanol at 4°C for another 24 h. Cells were stained with 50 μg/ml propidium iodide (PI) containing 400 U/ml RNase and detected using a FACSCalibur flow cytometer (BD Biosciences, CA, USA). Cell cycle distributions expressed by PI content were analyzed by FlowJo (FlowJo LLC, OR, USA) or ModFit LT software (Verity Software House, ME, USA).

### Cell Apoptosis Assay

Control or EXDFP knock-down SKOV3 cells (1 × 10^5^/ml) treated with or without various concentrations of paclitaxel were seeded into 6 well plates in a volume of 2 ml culture medium and cultured for 24 h. Cells were stained using an Annexin V and PI staining kit (V13242, Thermo Fisher Scientific, MA, USA) and detected on a FACSCalibur (BD Biosciences). Annexin V and/or PI positive apoptotic cells were analyzed using FlowJo software (FlowJo LLC, OR, USA).

### Xenograft Nude Mice Models

For the subcutaneous tumor model, 1 × 10^6^ IGROV1 control or EXDPF knock-down cells were injected subcutaneously into the right front flank of 6 - 8 weeks old BALB/c null nude mice. Each group contains 6 mice. Four weeks later, mice were sacrificed. Tumor nodes were removed, photographed and weighed.

For intraperitoneal metastasis tumor model, 1 × 10^6^ SKOV3 control or EXDPF knock-down cells were injected intraperitoneally into the abdomen of 6 - 8 weeks old BALB/c null nude mice. Each group contains 6 mice. In this model, tumor nodes could be formed in multiple abdominal organs or tissues, mainly in small intestinal, colon, mesenterium, liver and muscular tissues of abdominal cavity as showed by previous studies (34–38). Four weeks later, mice were sacrificed. All tumors nodes from abdominal organs or tissues were surgically removed, photographed, counted and weighed.

For the lung metastasis tumor model, 1 × 10^6^ SKOV3 control or EXDPF knock-down cells were injected into the tail vein of 6 - 8 weeks old BALB/c null nude mice. Each group contains 6 mice. Mice were sacrificed six weeks later. Lungs were removed, fixed with 4% paraformaldehyde, embedded in paraffin and cut into 5 μm slices follow by Haematoxylin Eosin (HE) staining. Tumor burdens were analyzed using ImageJ software and expressed as the percentage of areas of tumors to the whole lung.

### Statistical Analysis

All experiments were at least triplicated. Statistical data are shown as mean ± standard deviation (SD). Statistical differences between different groups were analyzed using GraphPad Prism 5.0 software (GraphPad Software Inc., CA, USA) by Student’s t-test for Gaussian distribution data and by Mann-Whitney nonparametric test for non-Gaussian distribution data. A *p* < 0.05 was considered that the difference was statistically significant.

## Results

### EXDPF Higher Expressed in Ovarian Tumors Compared to Normal Ovarian Tissue Controls

**Figure 1:**
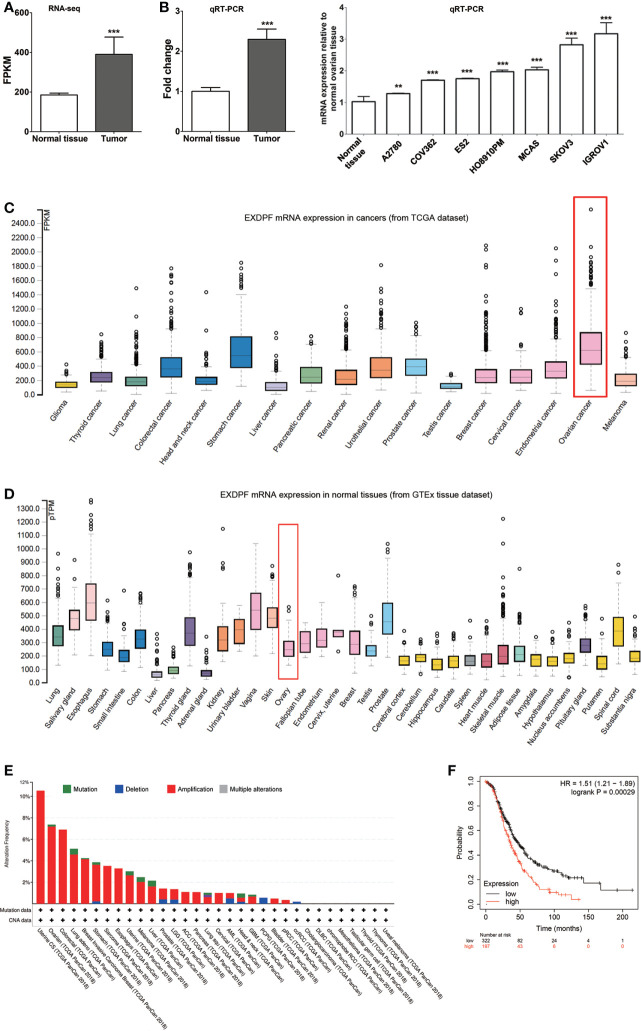
EXDPF over-expressed in ovarian tumors which correlated positively with poor OS of patients. **(A)** Total mRNA from three pairs of samples, both epithelial ovarian tumors and ovarian normal tissue controls from the same patients, were used for RNA sequencing. EXDPF mRNA expression levels were presented as fragments per kilobase of transcript per million mapped reads (FPKM). Statistical analysis was tested between ovarian tumors and normal tissues. **(B)** Eight pairs including the 3 pairs used for RNA sequencing of ovarian tumors and normal ovarian tissue controls were used for total RNA extraction followed by qRT-PCR assay. EXDPF expression levels were present as the ratio of Ct values of EXDPF to that of GAPDH. EXDPF expression levels of normal tissues were normalized to 1.0. Statistical analysis was tested between ovarian tumors and the normal tissue controls. **(C)** Total mRNA was extracted from seven epithelial ovarian cancer cell lines (SKOV3, IGROV1, MACS, HO8910PM, ES2, COV362 and A2780) and normal ovarian tissues as in panel **(B)** and detected by qRT-PCR assay as above. Statistical analysis was performed between ovarian cancer cell lines and the normal tissue controls. **(D)** EXDPF mRNA expression levels in ovarian tumors (upper panel) and normal ovarian tissues (lower panel) indexed from TCGA and GTEx database, respectively, were compared using The Human Protein Atlas online dataset. Rectangle marks ovarian cancer or normal ovarian tissue data. **(E)** cBioPortal online database was used to detect the DNA Copy Number Alteration (CNA) of EXDPF in 32 different human tumors. Data were indexed from TCGA database. Ovarian cancer had the second highest DNA amplification of EXDPF among these cancers tested. Red bar represents DNA amplification and green bar represents DNA mutation. **(F)** Kaplan Meier-Plotter online database was used to analyze the relationship between EXDPF mRNA expression levels and ovarian cancer patient overall survival (OS). Red line shows the samples with higher EXDPF expression levels while black line with lower EXDPF expression levels. Data are presented as mean ± SD. **p < 0.01; ***p < 0.001.

As ovarian cancer is highly heterogenicity, elucidation of novel genes involved in ovarian cancer development is critical for thoroughly understanding the underlying mechanisms of tumorigenesis. By using RNA sequencing, we found that EXDPF is higher expressed in ovarian tumors compared to normal ovarian tissues from the same 3 patients (**Figure 1A**). This result was confirmed by qRT-PCR in 8 pairs of samples (**Figure 1B**). Furthermore, we detected the EXDPF gene expression levels in several epithelial ovarian cancer cell lines by qRT-PCR. All tested 7 cell lines (A2780, COV362, ES2, HO8910PM, MACS, IGROV1 and SKOV3) expressed significantly higher EXDPF mRNA than normal ovarian tissues (**Figure 1C**). Using the Human Protein Atlas online database, this study showed that ovarian cancer had one of the highest mRNA expression levels of EXDPF among 17 tested tumors, while normal ovarian tissues had weak or low expression of EXDPF among 36 normal human tissues (**Figure 1D**). The cBioPortal online dataset contains DNA Copy Number Alteration (CNA) and mutation data of tumor samples from TCGA database. Here, we showed that EXDPF DNA is amplified in 7.2% tumors of ovarian cancer patients, which is the second highest proportion just lower than that of Uterine tumors of 10.5% (**Figure 1E**). Based on the above results, we conclude that EXDPF is general highly expressed in ovarian tumors. Then, we investigated the relationship between expression levels of EXDPF and ovarian cancer patient prognosis in Kaplan Meier-Plotter online database. Our study showed that higher expression of EXDPF is significantly correlated with shorter OS of ovarian cancer patients (**Figure 1F**).

### EXDPF Promotes Ovarian Cancer Tumorigenesis and Metastasis

**Figure 2:**
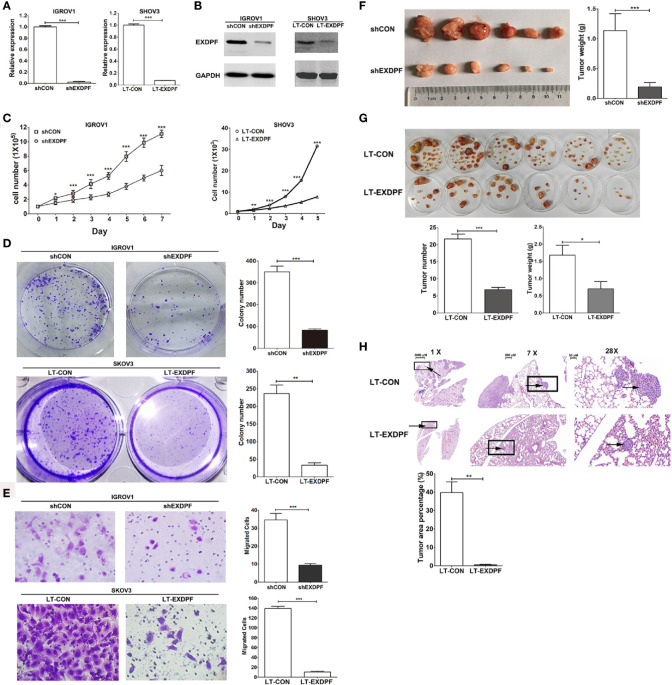
Knock-down of EXDPF inhibits ovarian cancer tumorigenesis. **(A)** EXDPF mRNA expression levels in IGROV1 (left panel) and SKOV3 (right panel) cells were knock-down by shRNA plasmid (shEXDPF) and lentivirus expressing shRNA targeting EXDPF (LT-EXDPF), respectively. IGROV1 transfected with control plasmid (shCON) and SKOV3 infected with control lentivirus (LT-CON) served as negative controls. **(B)** Decreased EXDPF protein levels caused by RNA interference in IGROV1 (left panel) and SKOV3 (right panel) cells were detected by Western Blotting. **(C)** EXDPF knock-down and control IGROV1 (left panel) or SKOV3 (right panel) cells were cultured and counted for successive 5 to 7 days. Cell numbers were compared between EXDPF knock-down and control groups at each day. **(D)** One thousand of IGROV1 and SKOV3 cells, both EXDPF normal expression control (shCON and LT-CON, respectively) or knock-down (shEXDPF and LT-EXDPF, respectively) cells, were seeded into 6 well plates and cultured for 10 days for colony counting. Representative images of colonies in a well of 6 well plates were shown in the left panels, and statistical data were shown in the right panels. **(E)** For detection of the ability of cell migration, 1 × 10^5^ EXDPF knock-down or control cells as elucidated in panel **(D)** were seeded into a well of transwells and plated into 24 well plates. Transferred cells were counted after a culture duration of 18 h. The left panels show the representative images and the right panels show the statistical data compared between EXDPF knock-down and control groups. Images were token by a microscope under 200-fold magnitude. **(F)** BALB/c null nude mice were subcutaneously inoculated with 1 × 10^6^ EXDPF knock-down (shEXDPF group) or control (shCON group) IGROV1 cells. Mice were sacrificed 4 weeks later and tumors were collected and weighed. Tumor images were shown in left panel. Statistical data of tumor weights were shown in the right panel. **(G)** EXDPF knock-down (LT-EXDPF group) or control (LT-CON group) SKOV3 cells were inoculated intraperitoneally into nude mice at the amount of 1 × 10^6^. Four weeks later, mice were sacrificed and tumor nodes from the abdominal organs or tissues of each mouse were removed to a 6 cm dish, photographed and weighed. Tumor images were shown in the upper panel while statistical analysis of tumor numbers and tumor weights were shown in the lower panels. **(H)** 1 × 10^6^ EXDPF knock-down (LT-EXDPF group) or control (LT-CON group) SKOV3 cells were injected into nude mice through tail vein. Six weeks later, mice were sacrificed and lungs were derived, fixed with 4% paraformaldehyde and sliced into 5 μm sections followed by HE staining. The upper left panel is the scan images of whole lungs. The upper middle and right panels are a 7- and 28-fold magnitude zoomed images of the rectangle marked areas in the left and middle panels, respectively. Arrows within each sample marked the same tumor. The tumor burden was defined as the percentage of tumor areas to the whole lung areas. Statistical analysis of tumor burdens between mice inoculated with EXDPF knock-down and control SKOV3 cells was shown in the lower panel. Statistic data are presented as mean ± SD. *p < 0.05; **p < 0.01; ***p < 0.001.

To investigate the role of EXDPF in ovarian cancer development, we knock-down EXDPF expression in two cell lines that have the highest mRNA expression levels of EXDPF among all cell lines tested in this study. A plasmid expressing shRNA targeting EXDPF was used for temporary knock-down of EXDPF in IGROV1 cells. And lentivirus expressing shRNA targeting EXDPF was used for permanently knock-down of EXDPF in SKOV3 cells. EXDPF mRNA expression levels were decreased more than 90% percent in both IGROV1 and SKOV3 cells (**Figure 2A**). The decrease of protein levels of EXDPF was validated by Western blotting (**Figure 2B**).

One of the key roles of cancer cells is the high ability of proliferation. So, we detected the effect of EXDPF knock-down on ovarian cancer cell proliferation. As shown in **Figure 2C**, knock-down of EXDPF significantly inhibited proliferation of both IGROV1 and SKOV3 cells. Also, the colony growth ability of IGROV1 and SKOV3 cells was tremendously inhibited by EXDPF knock-down (**Figure 2D**). Transwell assay was used to measure the effect of EXDPF on migratory ability of ovarian cancer cells. EXDPF knock-down largely hampered IGROV1 and SKOV3 cell migration (**Figure 2E**).

Xenograft in BALB/c null nude mice is a widely used animal model for investigation of tumor development *in vivo*. Here, we compared the tumor growth ability in nude mice of EXDPF knock-down and the control IGROV1 cells inoculated subcutaneously. As expected, the weights of tumors grown from EXDPF knock-down cells (0.19 ± 0.18 gram) were significantly decreased compared to that from control IGROV1 cells (1.14 ± 0.69 gram), *p* = 0.0003 (**Figure 2F**). Inoculating cancer cells intraperitoneally could, at least to some extent, mimic ovarian cancer growth and metastasis in abdomen. In this study, SKOV3 cells with or without EXDPF knock-down were injected into the abdominal cavity of nude mice. Four weeks later, all tumor nodes from the organs or tissues of abdomen were collected, counted and weighed. As shown in **Figure 2G**, knock-down of EXDPF in SKOV3 cells strongly decreased tumor counts (6.83 ± 2.23 *vs.* 25.00 ± 4.98, *p* < 0.0001) and weights (0.71 ± 0.51 gram *vs.* 1.68 ± 0.71 gram, *p* = 0.0152) in nude mice compared to the control cells. Injection of tumor cells into mice through the tail vein is a good model of lung metastasis. Here, one million EXDPF knock-down or control SKOV3 cells were injected intravenously into the nude mice. Six weeks later, mice were sacrificed and lungs were removed followed by HE staining for tumor nodes detection. Tumor burden, presented by the percentage of tumor area to the whole lung area, of mice inoculated with EXDPF knock-down SKOV3 cells was extremely lower than that of control mice (0.52 ± 0.37 *vs.* 39.71 ± 10.14, *p* = 0.0026) (**Figure 2H**).

### Knock-Down of EXDPF Sensitized SKOV3 Cells to Paclitaxel Therapy

**Figure 3:**
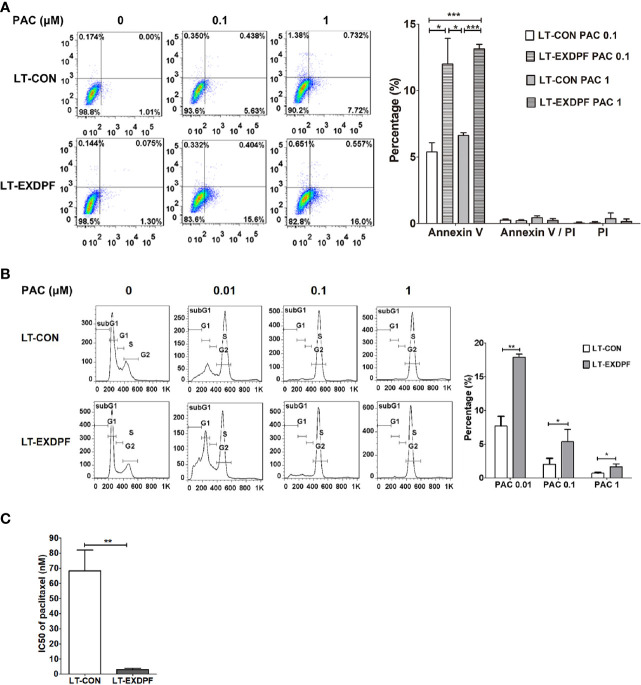
Knock-down of EXDPF sensitized SKOV3 cell to paclitaxel treatment. **(A)** EXDPF knock-down (LT-EXDPF group) or control (LT-CON group) SKOV3 cells were treated with or without 0.1 or 1 μM paclitaxel (PAC) for 24 h. Cell apoptosis was detected by Annexin V and PI staining assay. Annexin V and/or PI positive cells represent the apoptotic cells. Representative images are shown in the left panel and statistical data are shown in the right panel. LT-CON PAC 0.1: LT-CON cells treated with 0.1 μM paclitaxel. LT-EXDPF PAC 0.1: LT-EXDPF cells treated with 0.1 μM paclitaxel. LT-CON PAC 1: LT-CON cells treated with 1 μM paclitaxel. LT-EXDPF PAC 1: LT-EXDPF cells treated with 1 μM paclitaxel. **(B)** EXDPF knock-down (LT-EXDPF group) or control (LT-CON group) SKOV3 cells were treated with or without 0.01, 0.1 or 1 μM paclitaxel for 24 h. Cell cycle distribution was detected by PI staining. Representative data and statistical data are shown in the left and right panel, respectively. SubG1, subG1 apoptotic cell phase. G1: G0/G1 phase. S: S phase. G2: G2/M phase. PAC 0.01, PAC 0.1 and PAC 1: cells treated with paclitaxel at the concentration of 0.01, 0.1 and 1 μM, respectively. **(C)** A total volume of 100 μl culture medium contain 2 × 10^3^ EXDPF knock-down (LT-EXDPF group) or control (LT-CON group) SKOV3 cells were seeded into 96 well plates and treated with serial diluted paclitaxel at concentrations from 10,000 to 4.57 nM for 72 h. Cell viability was measured by MTS assay and IC_50_s were calculated. The statistical difference of IC50s between EXDPF knock-down (LT-EXDPF group) and control (LT-CON group) SKOV3 cells was analyzed. Statistic data are presented as mean ± SD. *p < 0.05; **p < 0.01; ***p < 0.001.

Synthetic lethality is an effective therapeutic strategy against cancer. Here, we detected the synthetic effect of EXDPF knock-down and paclitaxel chemotherapy. The data showed that when SKOV3 control cells treated alone with paclitaxel, there were only about 5% of Annexin V staining positive apoptotic cells at high dose (1 μM) of paclitaxel (**Figure 3A**). However, the apoptotic cells rose to more than 10% in EXDPF knock-down SKOV3 cells when treated with even low dose (0.1 μM) of paclitaxel (**Figure 3A**). PI staining showed more percentage of cells with DNA degradation in EXDPF knock-down SKOV3 cells treated with serial diluted paclitaxel from 1 μM to 0.01 μM compared to control cells (**Figure 3B**). We then hypothesized that EXDPF knock-down could decrease the IC_50_ of paclitaxel on SKOV3 cells. Indeed, the IC_50_ of paclitaxel on EXDPF knock-down SKOV3 cells is 3.03 nM which is 22.5-fold lower than that of 68.3 nM on SKOV3 control cells (**Figure 3C**). These data demonstrated the positive synthetic effect of EXDPF knock-down combined with paclitaxel treatment.

### EXDPF Promotes Ovarian Tumorigenesis Through Enhancing DNA Replication

**Figure 4:**
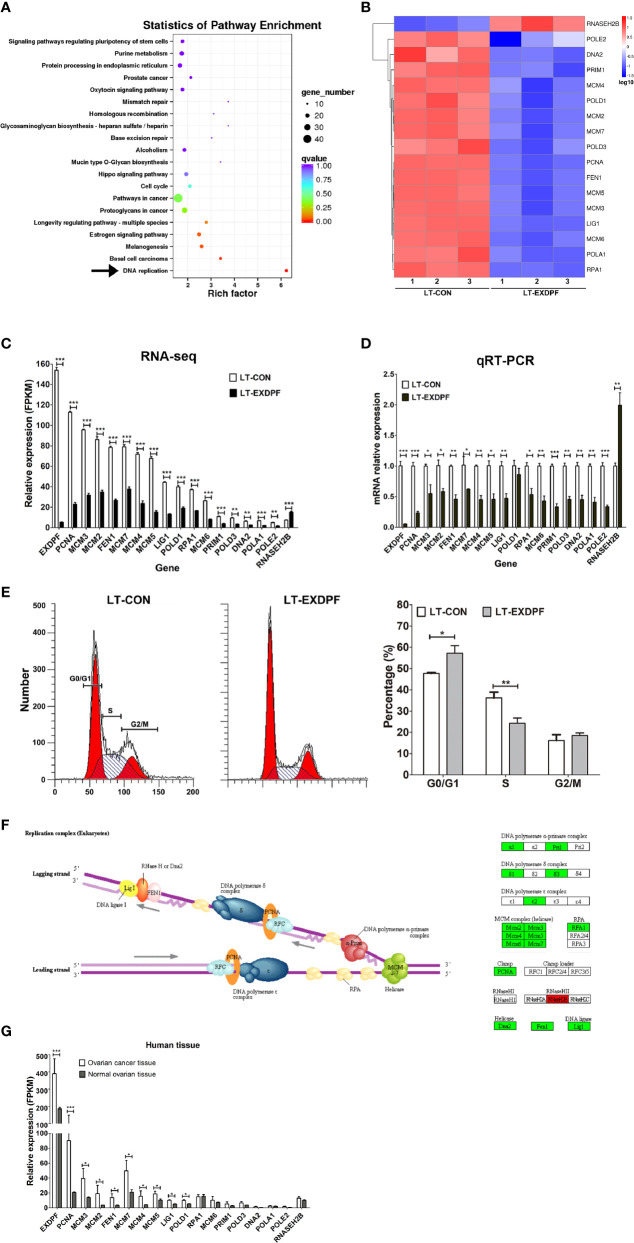
EXDPF promotes DNA replication in ovarian cancer. **(A)** Total mRNA from triplicated EXDPF knock-down (LT-EXDPF) and control (LT-CON) SKOV3 cells were used for whole genome mRNA sequencing. KEGG pathway enrichment shows the most significant altered pathways. Arrow points to the DNA replication pathway that has the lowest *p* value. **(B)** Heat map shows the 17 significantly altered genes enriched in DNA replication pathway. LT-CON: control SKOV3 cells. LT-EXDPF: EXDPF knock-down SKOV3 cells. **(C)** FPKM values indicate mRNA expression levels of the 17 significantly altered genes and EXDPF gene from the RNA sequencing assay. LT-CON: control SKOV3 cells. LT-EXDPF: EXDPF knock-down SKOV3 cells. **(D)** qRT-PCR was used to validate the mRNA expression of the 17 significantly altered genes and EXDPF gene in LT-EXDPF and LT-CON SKOV3 cells. Relative expression of genes in LT-CON control cells was normalized to 1.0. **(E)** PI staining was used to detect cell cycle distribution of LT-EXDPF and LT-CON SKOV3 cells. The left panel shows the representative data and the right panel shows the statistical analysis of differences between LT-EXDPF and LT-CON groups. G0/G1: G0/G1 phase. S: S phase. G2/M: G2/M phase. **(F)** Flowchart of genes affected by EXDPF knock-down in DNA replication pathway was derived from KEGG pathway enrichment analysis in panel **(A)**. The left panel shows the flowchart model of DNA replication. And the right panel shows genes involved in DNA replication. Green background indicates down-regulation while red background indicates up-regulation of mRNA expression levels of genes in SKOV3 cells upon EXDPF knock-down. **(G)** DNA replication pathway was up-regulated in ovarian cancer tissues compared to normal ovarian tissue controls. As indicated in **Figure 1A**, total mRNA from three pairs of samples, both epithelial ovarian tumors and ovarian normal tissue controls from the same patients, were used for RNA sequencing. mRNA expression levels of genes involving in DNA replication pathway were presented as fragments per kilobase of transcript per million mapped reads (FPKM). Statistical analysis was tested between ovarian cancer tissues and normal tissues. * *p* < 0.05; ** *p* < 0.01; *** *p* < 0.001.

To investigate the underlying mechanisms of EXDPF promoting ovarian cancer development, we used whole genome mRNA sequencing to compare the genomic mRNA expression pattern in EXDPF knock-down and control SKOV3 cells. KEGG pathway enrichment analysis showed that DNA replication pathway had the highest probability affected by EXDPF knock-down (**Figure 4A**). In DNA replication pathway, mRNA expression levels of 16 genes (PCNA, MCM3, MCM2, FEN1, MCM7, MCM4, MCM5, LIG1, POLD1, RPA1, MCM6, PRIM1, POLD3, DNA2, POLA1 and POLE2) were significantly decreased and 1 gene was increased (RNASEH2B) upon EXDPF knock-down (**Figures 4B, C**). We used qRT-PCR assay to validate the above 17 and EXDPF gene expression levels. The qRT-PCR experimental results were highly consistent with mRNA sequencing data (**Figure 4D**).

As knock-down of EXDPF highly decreased DNA replication pathway, which indicated DNA replication could be inhibited and cell cycle arrested upon lower expression of EXDPF. We detected cell cycle distribution of SKOV3 cells with or without EXDPF knock-down by FACS using PI staining. As expected, the percentage of EXDPF knock-down cells at S phase was significantly decreased while at G0/G1 phase was significantly increased (**Figure 4E**). Here, we show the summary flowchart of DNA replication pathway analyzed by KEGG pathway enrichment assay in **Figure 4F**. Also, we measured the expression levels of the above indicated 17 genes in human ovarian cancer tissues. As shown in **Figure 4G**, more than half of these genes are statistically over-expressed in ovarian cancer tissues compared to the normal ovarian tissue controls. In conclusion, these results demonstrated that knock-down of EXDPF significantly inhibited DNA replication in ovarian cancer cells.

## Discussion

An estimated about 62.6% - 66.0% ratio of death to incidence each year in ovarian cancer according to the GLOBOCAN reports, makes it the highest mortality rate and the fifth leading cause of death that accounts for 5% of death among gynecological malignancies (2, 4). Although novel therapeutic reagents, such as PARP inhibitors and anti-PD-1 and/or anti-PD-L1 antibodies, have been tested during these two decades, the five-year OS of ovarian cancer patients still just remains to 30 – 40 percent. One key cause that blocks developing novel efficient therapeutics to largely improve OS of ovarian cancer patients is the high heterogenicity of ovarian cancer, making it very hard to thoroughly uncover the underlying mechanisms associated with tumorigenesis. As ovary is an endocrine organ, we assume that dysfunction of endocrine could be associated with ovarian cancer tumorigenesis. EXDPF was demonstrated to promote the growth and differentiation of pancreatic exocrine glands while inhibit the growth and secretion function of endocrine glands in zebrafish (31). To measure the expression levels of EXDPF in ovarian cancer, 20 female patients with epithelial ovarian cancers were enrolled at the initial diagnosis. And only 8 patients got both tumor tissues and normal ovarian tissues. Pairs of tumor tissues and normal ovarian tissue controls from 3 out of these 8 patients were used for RNA-sequencing. All samples from these 8 patients were used for qRT-PCR to confirm the RNA-sequencing data. In this study, we showed that EXDPF is significantly higher expressed in ovarian tumors compared to normal ovarian tissues of the same patients. Consistent with our observation, a previous study showed high expression of EXDPF in hepatocellular carcinoma (39). The role of EXDPF in development of cancers especially ovarian cancer is still unknown. Except to exploring expression levels and functions of EXDPF by RNA-sequencing and qRT-PCR, public available databases containing a large number of samples were used to confirm our study results. We validated the high expression of EXDPF in ovarian tumors while low expression in normal ovarian tissues using the Human Protein Atlas dataset. Also, EXDPF DNA amplification could be detected in 7.2% ovarian tumors according to the cBioPortal online database. These results validated the high expression of EXDPF in ovarian cancer. To investigate the effect of EXDPF overexpression on clinical prognosis of ovarian cancer patients, we used the Kaplan Meier-Plotter online database to study the relationship of EXDPF expression levels to the OS of patients. The results showed that higher EXDPF expression correlated positively with poor OS of ovarian cancer patients. Together, these results demonstrated that EXDPF was over-expressed in ovarian tumors and correlated with poor OS of ovarian cancer patients.

As high EXDPF expression correlates with poor OS of ovarian cancer patients, it is meaningful to elucidate the functions of EXDPF in ovarian cancer. siRNA interference assays were used to knock-down EXDPF expression in IGROV1 and SKOV3 cells, which had the highest EXDPF mRNA expression levels among all tested cell lines in this study. EXDPF expression was knock-down temporarily in IGROV1 cells by shRNA expressing plasmid while permanently in SKOV3 cells by lentivirus expressing shRNA targeting EXDPF mRNA. By these methods, EXDPF mRNA expression levels were decreased more than 90% both in IGROV1 and SKOV3 cells. High abilities of proliferation, colony growth and migration are the basic properties of cancer cells. Our *in vitro* study showed that knock-down of EXDPF highly inhibited IGROV1 and/or SKOV3 cell proliferation, colony growth and migration. These data demonstrated EXDPF to be a pro-oncogene in ovarian cancer. Inoculation of cancer cells into BALB/c null nude mice to develop xenografts is a widely used assay to investigate the characteristics of cancer cells *in vivo*. We injected IGROV1 cells with or without knock-down of EXDPF subcutaneously into nude mice to measure tumor growth in mouse models. The results showed that knock-down of EXDPF significantly inhibited tumor growth. We also injected EXDPF knock-down or control SKOV3 cells into the abdominal cavity of nude mice to mimic human ovarian tumor growth and metastasis of advanced disease patients who usually accompanied with abdominal tumor metastasis. This model is widely adopted in ovarian cancer metastasis studies even though it cannot differentiate primary tumor cell dissemination from metastasis nodes (34–38). In this model, tumor node numbers correlated positively with migratory ability while total tumor weights correlated positively with proliferation ability of cancer cells. Our results showed that EXDPF knock-down strongly inhibited both tumor node numbers and tumor weights. Cancer cells injected into nude mice *via* tail vein is a widely used model to assess tumor growth and metastasis in the lungs of mice. Here, we also adopted this model to detect the effect of EXDPF knock-down on SKOV3 cells. Tumor burden presented as ratio of tumor areas to the whole lung areas correlated positively with cancer cell proliferation and migratory abilities. The results also showed that knock-down of EXDPF strongly inhibited tumor growth and metastasis in the lungs of nude mice. The above data demonstrated that EXDPF contributed to tumor growth and metastasis of ovarian cancer. Also, a previous study showed that circ-FOXM1 increased non-small cell lung cancer (NSCLC) cell proliferation and migration through upregulating the protein level of EXDPF and metastasis-associated in colon cancer 1 (MACC1) (40). That study showed that EXDPF involve in tumor development, while the functions of EXDPF in cancers were unclear. Then we wonder if knock-down of EXDPF could sensitize ovarian cancer cells to paclitaxel therapy. Here, we found that EXDPF knock-down SKOV3 cells exhibited higher percentage of Annexin V positive and DNA degraded apoptotic cells detected by FACS assay. The IC_50_ of paclitaxel in EXDPF knock-down SKOV3 cells was 22.5-fold lower than that of control cells. This means that combination of EXDPF knock-down and paclitaxel chemotherapy has positive synthetical antitumor efficacy. So, EXDPF may be a potential target candidate for ovarian cancer therapies.

To elucidate the underlying mechanisms of promoting ovarian cancer development by EXDPF, we sequenced mRNA from EXDPF knock-down and the control SKOV3 cells. KEGG pathway enrichment analysis showed that EXDPF knock-down mostly inhibited DNA replication pathway. Sixteen genes (PCNA, MCM3, MCM2, FEN1, MCM7, MCM4, MCM5, LIG1, POLD1, RPA1, MCM6, PRIM1, POLD3, DNA2, POLA1 and POLE2) were significantly decreased and 1 gene (RNASEH2B) increased in this pathway upon knock-down of EXDPF. It is rational that EXDPF knock-down could inhibit cell cycle progress by decrease DNA synthesis. As expected, EXDPF knock-down SKOV3 cells had higher percentage at G1 phase while lower percentage at S phase. Furthermore, using human ovarian cancer tissues and the normal ovarian tissues from the same patients, we demonstrated that DNA replication pathway associated genes were statistically over-expressed in ovarian tumors. In general, these results demonstrated that EXDPF mediated DNA replication in ovarian cancer.

## Conclusions

In summary, this study thoroughly demonstrated that EXDPF promoted ovarian cancer tumorigenesis and metastasis. The underlying mechanisms are associated with enhanced DNA replication induced by EXDPF. EXDPF could be a novel therapeutic target for ovarian cancer therapies.

## Data Availability Statement

The datasets presented in this study can be found in online repositories. The names of the repository/repositories and accession number(s) can be found below: https://www.ncbi.nlm.nih.gov/, PRJNA514879.

## Ethics Statement

This study was approved by and carried out in accordance with the recommendations of the Ethical Committee of Fengxian District Central Hospital. Written informed consents were given by all participants according to the Declaration of Helsinki. Animal studies were performed according to the care and use of animal guidelines and approved by the Animal Care and Use committee of the East China Normal University School of Life Sciences.

## Author Contributions

YX received the funding, and designed this project. YX, YXL, and YY performed the experiments and data analysis. PJ, YHL, CW, and RZ performed patient enrollment, sample collection and data analysis. RZ also helped in guiding this study. YX wrote the manuscript. All authors contributed to the article and approved the submitted version.

## Funding

This work was supported by the National Natural Science Foundation of China (Grant No. 81702563 to YX), and the Postdoctoral Science Foundation of China (Grant No. 2019M652850 to YX).

## Conflict of Interest

The authors declare that the research was conducted in the absence of any commercial or financial relationships that could be constructed as a potential conflict of interest.

## References

[1] Torre LA Bray F Siegel RL Ferlay J Lortet-Tieulent J Jemal A. Global Cancer Statistics, 2012. CA Cancer J Clin (2015) 65:87–108. 10.3322/caac.21262 25651787

[2] Siegel RL Miller KD Jemal A. Cancer Statistics, 2018. CA Cancer J Clin (2018) 68:7–30. 10.3322/caac.21442 29313949

[3] Li X Tang M Zhu Q Wang X Lin Y Wang X. The Exosomal Integrin alpha5beta1/AEP Complex Derived From Epithelial Ovarian Cancer Cells Promotes Peritoneal Metastasis Through Regulating Mesothelial Cell Proliferation and Migration. Cell Oncol (Dordr) (2020) 43:263–77. 10.1007/s13402-019-00486-4 PMC12990696 32080801

[4] Sung H Ferlay J Siegel RL Laversanne M Soerjomataram I Jemal A. Global Cancer Statistics 2020: GLOBOCAN Estimates of Incidence and Mortality Worldwide for 36 Cancers in 185 Countries. CA Cancer J Clin (2021) 0:1–41. 10.3322/caac.21660 33538338

[5] Colombo N Lorusso D Scollo P. Impact of Recurrence of Ovarian Cancer on Quality of Life and Outlook for the Future. Int J Gynecol Cancer (2017) 27:1134–40. 10.1097/IGC.0000000000001023 PMC5499966 28640766

[6] Agarwal R Kaye SB. Ovarian Cancer: Strategies for Overcoming Resistance to Chemotherapy. Nat Rev Cancer (2003) 3:502–16. 10.1038/nrc1123 12835670

[7] Ushijima K. Treatment for Recurrent Ovarian Cancer-At First Relapse. J Oncol (2010) 2010:497429. 10.1155/2010/497429 20066162 PMC2801501

[8] Damia G Broggini M. Platinum Resistance in Ovarian Cancer: Role of DNA Repair. Cancers (Basel) (2019) 11:1–15. 10.3390/cancers11010119 PMC6357127 30669514

[9] Mirza MR Pignata S Ledermann JA. Latest Clinical Evidence and Further Development of PARP Inhibitors in Ovarian Cancer. Ann Oncol (2018) 29:1366–76. 10.1093/annonc/mdy174 29750420

[10] Mirza MR Coleman RL Gonzalez-Martin A Moore KN Colombo N Ray-Coquard I. The Forefront of Ovarian Cancer Therapy: Update on PARP Inhibitors. Ann Oncol (2020) 31:1148–59. 10.1016/j.annonc.2020.06.004 32569725

[11] Walsh CS. Latest Clinical Evidence of Maintenance Therapy in Ovarian Cancer. Curr Opin Obstet Gynecol (2020) 32:15–21. 10.1097/GCO.0000000000000592 31833941

[12] Boussios S Moschetta M Karihtala P Samartzis EP Sheriff M Pappas-Gogos G. Development of New Poly(ADP-Ribose) Polymerase (PARP) Inhibitors in Ovarian Cancer: Quo Vadis? Ann Transl Med (2020) 8:1706. 10.21037/atm.2020.03.156 33490218 PMC7812175

[13] Ledermann J Harter P Gourley C Friedlander M Vergote I Rustin G. Olaparib Maintenance Therapy in Patients With Platinum-Sensitive Relapsed Serous Ovarian Cancer: A Preplanned Retrospective Analysis of Outcomes by BRCA Status in a Randomised Phase 2 Trial. Lancet Oncol (2014) 15:852–61. 10.1016/S1470-2045(14)70228-1 24882434

[14] Pujade-Lauraine E Ledermann JA Selle F Gebski V Penson RT Oza AM. Olaparib Tablets as Maintenance Therapy in Patients With Platinum-Sensitive, Relapsed Ovarian Cancer and a BRCA1/2 Mutation (SOLO2/ENGOT-Ov21): A Double-Blind, Randomised, Placebo-Controlled, Phase 3 Trial. Lancet Oncol (2017) 18:1274–84. 10.1016/S1470-2045(17)30469-2 28754483

[15] Arend R Westin SN Coleman RL. Decision Analysis for Secondline Maintenance Treatment of Platinum Sensitive Recurrent Ovarian Cancer: A Review. Int J Gynecol Cancer (2020) 30:684–94. 10.1136/ijgc-2019-001041 32079709

[16] Poveda A Floquet A Ledermann JA Asher R Penson RT Oza AM. Final Overall Survival (OS) Results From SOLO2/ENGOT-Ov21: A Phase III Trial Assessing Maintenance Olaparib in Patients (Pts) With Platinum-Sensitive, Relapsed Ovarian Cancer and a BRCA Mutation. J Clin Oncol (2020) 38:6002–2. 10.1200/JCO.2020.38.15_suppl.6002

[17] Ning F Cole CB Annunziata CM. Driving Immune Responses in the Ovarian Tumor Microenvironment. Front Oncol (2020) 10:604084. 10.3389/fonc.2020.604084 33520713 PMC7843421

[18] Brahmer JR Tykodi SS Chow LQ Hwu WJ Topalian SL Hwu P. Safety and Activity of Anti-PD-L1 Antibody in Patients With Advanced Cancer. N Engl J Med (2012) 366:2455–65. 10.1056/NEJMoa1200694 PMC3563263 22658128

[19] Gaillard SL Secord AA Monk B. The Role of Immune Checkpoint Inhibition in the Treatment of Ovarian Cancer. Gynecol Oncol Res Pract (2016) 3:11. 10.1186/s40661-016-0033-6 27904752 PMC5122024

[20] Torre LA Trabert B Desantis CE Miller KD Samimi G Runowicz CD. Ovarian Cancer Statistics, 2018. CA Cancer J Clin (2018) 68:284–96. 10.3322/caac.21456 PMC6621554 29809280

[21] Kroeger PT Jr Drapkin R. Pathogenesis and Heterogeneity of Ovarian Cancer. Curr Opin Obstet Gynecol (2017) 29:26–34. 10.1097/GCO.0000000000000340 27898521 PMC5201412

[22] Reid BM Permuth JB Sellers TA. Epidemiology of Ovarian Cancer: A Review. Cancer Biol Med (2017) 14:9–32. 10.20892/j.issn.2095-3941.2016.0084 28443200 PMC5365187

[23] Cancer Genome Atlas Research N. Integrated Genomic Analyses of Ovarian Carcinoma. Nature (2011) 474:609–15. 10.1038/nature10166 PMC3163504 21720365

[24] Balendran S Liebmann-Reindl S Berghoff AS Reischer T Popitsch N Geier CB. Next-Generation Sequencing-Based Genomic Profiling of Brain Metastases of Primary Ovarian Cancer Identifies High Number of BRCA-Mutations. J Neurooncol (2017) 133:469–76. 10.1007/s11060-017-2459-z PMC5537326 28497333

[25] Ahmed AA Etemadmoghadam D Temple J Lynch AG Riad M Sharma R. Driver Mutations in TP53 are Ubiquitous in High Grade Serous Carcinoma of the Ovary. J Pathol (2010) 221:49–56. 10.1002/path.2696 20229506 PMC3262968

[26] Ramalingam P. Morphologic, Immunophenotypic, and Molecular Features of Epithelial Ovarian Cancer. Oncology (Williston Park) (2016) 30:166–76. 26892153

[27] Perren TJ. Mucinous Epithelial Ovarian Carcinoma. Ann Oncol (2016) 27 Suppl 1:i53–7. 10.1093/annonc/mdw087 27141073

[28] Meinhold-Heerlein I Hauptmann S. The Heterogeneity of Ovarian Cancer. Arch Gynecol Obstet (2014) 289:237–9. 10.1007/s00404-013-3114-3 24318356

[29] Kobel M Kalloger SE Boyd N Mckinney S Mehl E Palmer C. Ovarian Carcinoma Subtypes are Different Diseases: Implications for Biomarker Studies. PloS Med (2008) 5:e232. 10.1371/journal.pmed.0050232 19053170 PMC2592352

[30] Sieh W Kobel M Longacre TA Bowtell DD Defazio A Goodman MT. Hormone-Receptor Expression and Ovarian Cancer Survival: An Ovarian Tumor Tissue Analysis Consortium Study. Lancet Oncol (2013) 14:853–62. 10.1016/S1470-2045(13)70253-5 PMC4006367 23845225

[31] Jiang Z Song J Qi F Xiao A An X Liu NA. Exdpf is a Key Regulator of Exocrine Pancreas Development Controlled by Retinoic Acid and ptf1a in Zebrafish. PloS Biol (2008) 6:e293. 10.1371/journal.pbio.0060293 19067490 PMC2586380

[32] Xiao Y Yu Y Gao D Jin W Jiang P Li Y. Inhibition of CDC25B With WG-391D Impedes the Tumorigenesis of Ovarian Cancer. Front Oncol (2019) 9:236. 10.3389/fonc.2019.00236 31024841 PMC6463794

[33] Gyorffy B Lanczky A Szallasi Z. Implementing an Online Tool for Genome-Wide Validation of Survival-Associated Biomarkers in Ovarian-Cancer Using Microarray Data From 1287 Patients. Endocr Relat Cancer (2012) 19:197–208. 10.1530/ERC-11-0329 22277193

[34] Xiao Y Yu Y Jiang P Li Y Wang C Zhang R. The PI3K/mTOR Dual Inhibitor GSK458 Potently Impedes Ovarian Cancer Tumorigenesis and Metastasis. Cell Oncol (2020) 43:669–80. 10.1007/s13402-020-00514-8 PMC12990695 32382996

[35] Yoshida H Cheng W Hung J Montell D Geisbrecht E Rosen D. Lessons From Border Cell Migration in the Drosophila Ovary: A Role for Myosin VI in Dissemination of Human Ovarian Cancer. Proc Natl Acad Sci USA (2004) 101:8144–9. 10.1073/pnas.0400400101 PMC419571 15146066

[36] Sale S Orsulic S. Models of Ovarian Cancer Metastasis: Murine Models. Drug Discov Today Dis Models (2006) 3:149–54. 10.1016/j.ddmod.2006.05.006 PMC2662599 19337569

[37] Chen L Park SM Tumanov AV Hau A Sawada K Feig C. CD95 Promotes Tumour Growth. Nature (2010) 465:492–6. 10.1038/nature09075 PMC2879093 20505730

[38] Eckert MA Coscia F Chryplewicz A Chang JW Hernandez KM Pan S. Proteomics Reveals NNMT as a Master Metabolic Regulator of Cancer-Associated Fibroblasts. Nature (2019) 569:723–8. 10.1038/s41586-019-1173-8 PMC6690743 31043742

[39] Mao Z Li X Ma X Wang X Zhang J Fan X. Pancreatic Progenitor Cell Differentiation and Proliferation Factor Predicts Poor Prognosis in Heptaocellular Carcinoma. Medicine (Baltimore) (2019) 98:e14552. 10.1097/MD.0000000000014552 30817571 PMC6831259

[40] Liu G Shi H Deng L Zheng H Kong W Wen X. Circular RNA circ-FOXM1 Facilitates Cell Progression as ceRNA to Target PPDPF and MACC1 by Sponging miR-1304-5p in non-Small Cell Lung Cancer. Biochem Biophys Res Commun (2019) 513:207–12. 10.1016/j.bbrc.2019.03.213 30954221

