# Supplementary material for: The Exocrine Differentiation and Proliferation Factor (EXDPF) Gene Promotes Ovarian Cancer Tumorigenesis by Up-Regulating DNA Replication Pathway
Source: Front Oncol. 2021 May 10;11:669603. doi: 10.3389/fonc.2021.669603 (PMC8141798; doi:10.3389/fonc.2021.669603)
Supplement: Supplementary file 1 [file Table_1.docx]

Supplementary Material

# Supplementary Tables

**Table S1. Characteristics of ovarian cancer participants.**

|  | Tumor type | Tumor stage | Nationality | Age (year) |
| --- | --- | --- | --- | --- |
| CZ001 | Clear cell carcinoma | ⅠC | Chinese Han | 48 |
| CZ003 | Endometrioid carcinoma | ⅢC | Chinese Han | 55 |
| CZ004 | Serous carcinoma | ⅢA | Chinese Han | 50 |
| CZ008 | Poorly differentiated carcinoma | ⅣB | Chinese Han | 63 |
| CZ009 | Clear cell carcinoma | ⅣB | Chinese Han | 72 |
| FX002 | Clear cell carcinoma | ⅡB | Chinese Han | 63 |
| HFZ002 | Serous carcinoma |  | Chinese Han |  |
| HFZ003 | Serous carcinoma |  | Chinese Han |  |

**Table S2. Primers for qRT-PCR.**

| Gene | Forward primer (5'to3') | Revers primer (5'to3') |
| --- | --- | --- |
| EXDPF | CAAGCTAAAGCATGGCGGC | TGTTGCTGGAAGTGGAACCC |
| GAPDH | ACCCAGAAGACTGTGGATGG | TTCAGCTCAGGGATGACCTT |
| DNA2 | GGTGCCATACCTGTCACAAAT | AGGACCGACAAGTTTCTGTCTA |
| FEN1 | CACCTGATGGGCATGTTCTAC | CTCGCCTGACTTGAGCTGT |
| LIG1 | ACAGTTCCCCATCAGGGATTC | CTCTGTGAGGCTTTCTTTCGG |
| MCM2 | ATGGCGGAATCATCGGAATCC | GGTGAGGGCATCAGTACGC |
| MCM3 | GGCCTCCATTGATGCTACCTA | ACTTTGGGACGAACTAGAGAACA |
| MCM4 | TGAACCTCTATACATGCAACGAC | CAGGGTAACGGTCAAAGAAGATT |
| MCM5 | AGCATTCGTAGCCTGAAGTCG | CGGCACTGGATAGAGATGCG |
| MCM6 | TCGGGCCTTGAAAACATTCGT | TGTGTCTGGTAGGCAGGTCTT |
| MCM7 | GCCTGTGGGAAATATCCCTCG | GTACCACCTGTCGGAACCC |
| PCNA | CCTGCTGGGATATTAGCTCCA | CAGCGGTAGGTGTCGAAGC |
| POLA1 | AGAAGCTCGCAGTGACAAAAC | AGGTGGTGGAGTTATTTGAGGT |
| POLD1 | CAGTGCCAAGGTGGTGTATGG | CTTGCTGATAAGCAGGTATGGG |
| POLD3 | GAGTTCGTCACGGACCAAAAC | GCCAGACACCAAGTAGGTAAC |
| POLE2 | ATTTACTCCTCCGGTGATAGGTT | GCATCTCCGATTTTGGTTGTACT |
| PRIM1 | ACATTCGCTACCAATCCTTCAAC | AGCTCCCAGCTTCACTGTATT |
| RNASEH2B | TAACCCCTGTTCAGGAGAAGG | ACACGTTATCCACCACAACTTG |
| RPA1 | CGGGAATGGGTTCTACTGTTTC | CGAGCACAAATGGTCCACTTG |
| FTH1 | ACTGGAACTGCACAAACTGG | ATCTTGCGCAAGTTGGTCAC |
